# 8-Bromo-3-(4-ethyl­phen­yl)-1-phenyl-3,3a,4,9b-tetra­hydro-1*H*-chromeno[4,3-*c*]isoxazole-3a-carbo­nitrile

**DOI:** 10.1107/S1600536813023982

**Published:** 2013-09-12

**Authors:** J. Kanchanadevi, G. Anbalagan, J. Srinivasan, M. Bakthadoss, B. Gunasekaran, V. Manivannan

**Affiliations:** a Department of Physics, Velammal Institute of Technology, Panchetty, Chennai 601204, India; b Department of Physics, Presidency College (Autonomous), Chennai 600 005, India; c Department of Organic Chemistry, University of Madras, Guindy Campus, Chennai 600 025, India; d Department of Physics & Nano Technology, SRM University, SRM Nagar, Kattankulathur, Kancheepuram District, Chennai 603 203, Tamil Nadu, India; e Department of Research and Development, PRIST University, Vallam, Thanjavur 613 403, Tamil Nadu, India

## Abstract

In the title compound, C_25_H_21_BrN_2_O_2_, the fused isoxazolidine ring adopts an envelope conformation with the N atom at the flap and the mean plane of the ring makes dihedral angles of 54.37 (12) and 87.32 (13)°, respectively, with the adjacent phenyl and benzene rings. The tetra­hydro­pyran ring has a half-chair conformation. In the crystal, mol­ecules are linked into a double-column structure along the *b*-axis direction through weak C—H⋯O and C—H⋯π inter­actions.

## Related literature

For the biological activity of cyano­acrylates, see: Zhang *et al.* (2009 ▶); Obniska *et al.* (2005 ▶). For related structures, see: Ye *et al.* (2009 ▶); Suresh *et al.* (2012 ▶); Kanchanadevi *et al.* (2013 ▶).
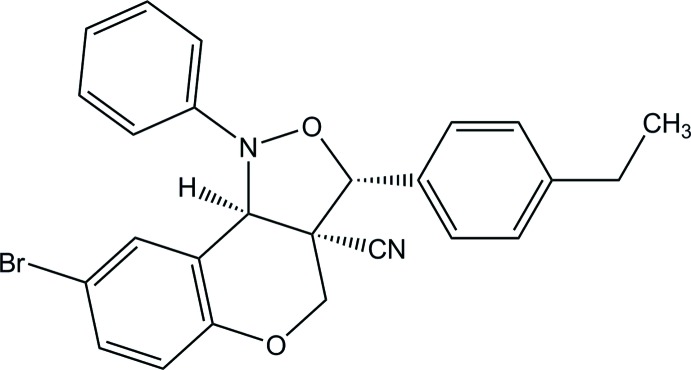


## Experimental

### 

#### Crystal data

C_25_H_21_BrN_2_O_2_
*M* *_r_* = 461.35
Triclinic, 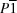

*a* = 9.8813 (3) Å
*b* = 9.9921 (3) Å
*c* = 11.1587 (3) Å
α = 94.125 (2)°
β = 92.196 (2)°
γ = 101.500 (2)°
*V* = 1075.27 (5) Å^3^
*Z* = 2
Mo *K*α radiation
μ = 1.94 mm^−1^
*T* = 295 K
0.25 × 0.20 × 0.15 mm

#### Data collection

Bruker APEXII CCD diffractometer
Absorption correction: multi-scan (*SADABS*; Sheldrick, 1996 ▶) *T*_min_ = 0.620, *T*_max_ = 0.748
27480 measured reflections
6895 independent reflections
4405 reflections with *I* > 2σ(*I*)
*R*_int_ = 0.031

#### Refinement

*R*[*F* ^2^ > 2σ(*F* ^2^)] = 0.044
*wR*(*F* ^2^) = 0.123
*S* = 1.02
6895 reflections
274 parameters
H-atom parameters constrained
Δρ_max_ = 0.54 e Å^−3^
Δρ_min_ = −0.81 e Å^−3^

### 

Data collection: *APEX2* (Bruker, 2008 ▶); cell refinement: *SAINT* (Bruker, 2008 ▶); data reduction: *SAINT*; program(s) used to solve structure: *SHELXS97* (Sheldrick, 2008 ▶); program(s) used to refine structure: *SHELXL97* (Sheldrick, 2008 ▶); molecular graphics: *PLATON* (Spek, 2009 ▶); software used to prepare material for publication: *SHELXL97*.

## Supplementary Material

Crystal structure: contains datablock(s) I. DOI: 10.1107/S1600536813023982/is5299sup1.cif

Structure factors: contains datablock(s) I. DOI: 10.1107/S1600536813023982/is5299Isup2.hkl

Supplementary material file. DOI: 10.1107/S1600536813023982/is5299Isup3.cml

Additional supplementary materials:  crystallographic information; 3D view; checkCIF report

## Figures and Tables

**Table 1:** Hydrogen-bond geometry (Å, °) *Cg*3, *Cg*4 and *Cg*5 are the centroids of the C1–C4/C8/C9, C10–C15 and C17–C22 rings, respectively.

*D*—H⋯*A*	*D*—H	H⋯*A*	*D*⋯*A*	*D*—H⋯*A*
C11—H11⋯O1^i^	0.93	2.57	3.475 (2)	165
C3—H3⋯*Cg*5^ii^	0.93	2.78	3.491 (3)	134
C5—H5*B*⋯*Cg*3^i^	0.97	2.86	3.730 (4)	149
C22—H22⋯*Cg*4^iii^	0.93	2.95	3.628 (3)	131

Symmetry codes: (i) 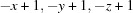; (ii) 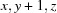; (iii) 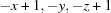.

**Fig. 1.**
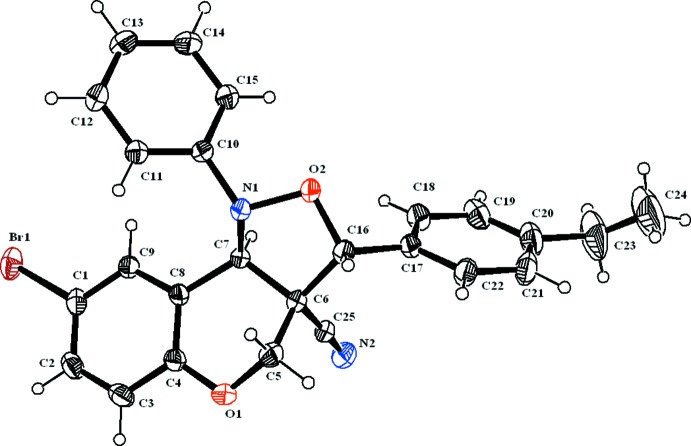
The molecular structure of the title compound, with atom labels and 30% probability displacement ellipsoids for non-H atoms.

**Fig. 2.**
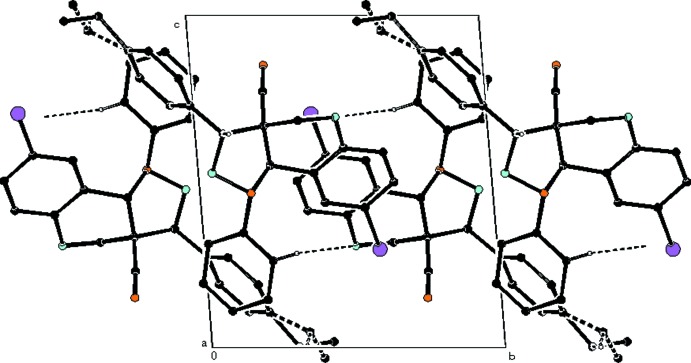
A packing diagram of the title compound, viewed down the a axis. Hydrogen bonds are shown as dashed lines.

## supplementary crystallographic information

### 1. Comment

Cyanoacrylates and its derivatives have been widely used as agrochemicals (Zhang *et al.*, 2009) and an important intermediate in drugs synthesis (Obniska *et al.*, 2005).

The geometric parameters of the title molecule (Fig. 1) agree well with reported similar structures (Ye *et al.*, 2009; Suresh *et al.*, 2012; Kanchanadevi *et al.*, 2013). The crystal packing is controlled by weak intermolecular C—H···O and C—H···π (C3—H3···*Cg*5^ii^, C5—H5B···*Cg*3^i^ and C22—H22···*Cg*4^iii^; symmetry codes as in Table 1; *Cg*3, *Cg*4 and *Cg*5 are the centroids of the rings defined by the atoms C1–C4/C8/C9, C10–C15 and C17–C22, respectively) interactions.

### 2. Experimental

A mixture of (*E*)-2-[(4-bromo-2-formylphenoxy)methyl]-3-(4-ethylphenyl)acrylonitrile (2 mmol, 0.75 g) and *N*-phenylhydroxylamine (3 mmol, 0.33 g) in ethanol (10 ml) was refluxed for 6 h. After the completion of the reaction as indicated by TLC, the reaction mixture was concentrated and the resulting crude mass was diluted with water (15 ml) and extracted with ethyl acetate (3 × 15 ml). The combined organic layer was washed with brine (3 × 15 ml) and dried over anhydrous Na_2_SO_4_, solvent was removed under reduced pressure. The crude mass was purified by column chromatography on silica gel (Acme 100–200 mesh), using ethyl acetate–hexane (1:9) to afford the pure compound as a colourless solid in 82% yield and melting point 171–173 °C.

### 3. Refinement

H atoms were positioned geometrically (C—H = 0.93–0.98 Å) and refined using riding model with *U*_iso_(H) = 1.2*U*_eq_(C) or 1.5*U*_eq_(C_methyl_).

### Crystal data

**Table:**

C_25_H_21_BrN_2_O_2_	*Z* = 2
*M**_r_* = 461.35	*F*(000) = 472
Triclinic, *P*1	*D*_x_ = 1.425 Mg m^−^^3^
Hall symbol: -P 1	Mo *K*α radiation, λ = 0.71073 Å
*a* = 9.8813 (3) Å	Cell parameters from 4405 reflections
*b* = 9.9921 (3) Å	θ = 2.1–31.1°
*c* = 11.1587 (3) Å	µ = 1.94 mm^−^^1^
α = 94.125 (2)°	*T* = 295 K
β = 92.196 (2)°	Block, colourless
γ = 101.500 (2)°	0.25 × 0.20 × 0.15 mm
*V* = 1075.27 (5) Å^3^	

### Data collection

**Table:**

Bruker APEXII CCD diffractometer	6895 independent reflections
Radiation source: fine-focus sealed tube	4405 reflections with *I* > 2σ(*I*)
Graphite monochromator	*R*_int_ = 0.031
Detector resolution: 0 pixels mm^-1^	θ_max_ = 31.1°, θ_min_ = 2.1°
ω and φ scans	*h* = −13→14
Absorption correction: multi-scan (*SADABS*; Sheldrick, 1996)	*k* = −14→14
*T*_min_ = 0.620, *T*_max_ = 0.748	*l* = −16→15
27480 measured reflections	

### Refinement

**Table:**

Refinement on *F*^2^	Primary atom site location: structure-invariant direct methods
Least-squares matrix: full	Secondary atom site location: difference Fourier map
*R*[*F*^2^ > 2σ(*F*^2^)] = 0.044	Hydrogen site location: inferred from neighbouring sites
*wR*(*F*^2^) = 0.123	H-atom parameters constrained
*S* = 1.02	*w* = 1/[σ^2^(*F*_o_^2^) + (0.0578*P*)^2^ + 0.3505*P*] where *P* = (*F*_o_^2^ + 2*F*_c_^2^)/3
6895 reflections	(Δ/σ)_max_ = 0.001
274 parameters	Δρ_max_ = 0.54 e Å^−^^3^
0 restraints	Δρ_min_ = −0.81 e Å^−^^3^

### Fractional atomic coordinates and isotropic or equivalent isotropic displacement parameters (Å^2^)

**Table:**

	*x*	*y*	*z*	*U*_iso_*/*U*_eq_	
Br1	0.04591 (3)	0.59905 (3)	0.29647 (2)	0.06392 (11)	
O1	0.42095 (16)	0.48260 (14)	0.69339 (13)	0.0480 (4)	
O2	0.32836 (18)	0.04730 (14)	0.52461 (12)	0.0479 (4)	
N1	0.33533 (18)	0.17431 (16)	0.46483 (14)	0.0387 (4)	
C6	0.3363 (2)	0.23582 (19)	0.66704 (16)	0.0366 (4)	
C8	0.2571 (2)	0.39431 (18)	0.52539 (16)	0.0350 (4)	
N2	0.1838 (2)	0.2514 (2)	0.85005 (17)	0.0566 (5)	
C7	0.2565 (2)	0.24777 (18)	0.54874 (15)	0.0337 (4)	
H7	0.1611	0.1965	0.5498	0.040*	
C16	0.3846 (2)	0.0964 (2)	0.64292 (17)	0.0417 (4)	
H16	0.4857	0.1146	0.6425	0.050*	
C10	0.2740 (2)	0.13817 (19)	0.34525 (16)	0.0366 (4)	
C12	0.2584 (2)	0.2032 (2)	0.14426 (18)	0.0490 (5)	
H12	0.2816	0.2674	0.0883	0.059*	
C17	0.3385 (2)	−0.0105 (2)	0.72803 (18)	0.0422 (4)	
C9	0.1687 (2)	0.4248 (2)	0.43612 (17)	0.0390 (4)	
H9	0.1143	0.3544	0.3861	0.047*	
C5	0.4613 (2)	0.3539 (2)	0.68345 (19)	0.0449 (5)	
H5A	0.5157	0.3445	0.7554	0.054*	
H5B	0.5188	0.3499	0.6153	0.054*	
C22	0.4339 (3)	−0.0410 (2)	0.81004 (19)	0.0512 (5)	
H22	0.5263	0.0027	0.8100	0.061*	
C25	0.2495 (2)	0.2419 (2)	0.77047 (17)	0.0408 (4)	
C4	0.3342 (2)	0.50191 (19)	0.60093 (17)	0.0391 (4)	
C15	0.1848 (2)	0.0155 (2)	0.31184 (19)	0.0480 (5)	
H15	0.1581	−0.0473	0.3684	0.058*	
C13	0.1712 (3)	0.0798 (3)	0.1099 (2)	0.0544 (6)	
H13	0.1368	0.0599	0.0305	0.065*	
C11	0.3113 (2)	0.2318 (2)	0.26118 (17)	0.0419 (4)	
H11	0.3723	0.3142	0.2836	0.050*	
C14	0.1354 (3)	−0.0133 (2)	0.1927 (2)	0.0570 (6)	
H14	0.0772	−0.0970	0.1691	0.068*	
C3	0.3218 (2)	0.6360 (2)	0.5880 (2)	0.0484 (5)	
H3	0.3717	0.7065	0.6406	0.058*	
C21	0.3925 (3)	−0.1363 (3)	0.8921 (2)	0.0663 (7)	
H21	0.4575	−0.1547	0.9477	0.080*	
C2	0.2369 (3)	0.6649 (2)	0.4985 (2)	0.0509 (5)	
H2	0.2297	0.7550	0.4893	0.061*	
C18	0.2022 (3)	−0.0779 (2)	0.7290 (2)	0.0526 (5)	
H18	0.1369	−0.0588	0.6741	0.063*	
C1	0.1616 (2)	0.5591 (2)	0.42159 (18)	0.0435 (5)	
C19	0.1623 (3)	−0.1735 (3)	0.8109 (3)	0.0635 (7)	
H19	0.0701	−0.2179	0.8105	0.076*	
C20	0.2571 (4)	−0.2044 (3)	0.8934 (3)	0.0696 (8)	
C23	0.2127 (6)	−0.3092 (5)	0.9833 (4)	0.1272 (18)	
H23A	0.2132	−0.2599	1.0615	0.153*	
H23B	0.1177	−0.3540	0.9615	0.153*	
C24	0.2878 (8)	−0.4092 (5)	0.9959 (6)	0.176 (3)	
H24A	0.2747	−0.4704	0.9241	0.265*	
H24B	0.2573	−0.4596	1.0633	0.265*	
H24C	0.3841	−0.3678	1.0091	0.265*	

### Atomic displacement parameters (Å^2^)

**Table:**

	*U*^11^	*U*^22^	*U*^33^	*U*^12^	*U*^13^	*U*^23^
Br1	0.0752 (2)	0.07064 (18)	0.05709 (16)	0.03362 (14)	0.00511 (12)	0.02685 (12)
O1	0.0583 (9)	0.0377 (7)	0.0422 (8)	−0.0007 (6)	−0.0080 (7)	−0.0010 (6)
O2	0.0789 (11)	0.0361 (7)	0.0330 (7)	0.0220 (7)	−0.0028 (7)	0.0050 (5)
N1	0.0537 (10)	0.0346 (8)	0.0295 (7)	0.0124 (7)	0.0001 (7)	0.0047 (6)
C6	0.0455 (11)	0.0356 (9)	0.0288 (8)	0.0079 (8)	−0.0005 (7)	0.0055 (7)
C8	0.0429 (10)	0.0304 (8)	0.0320 (8)	0.0066 (7)	0.0053 (7)	0.0049 (7)
N2	0.0735 (14)	0.0594 (12)	0.0382 (9)	0.0142 (10)	0.0083 (9)	0.0069 (8)
C7	0.0412 (10)	0.0300 (8)	0.0291 (8)	0.0052 (7)	0.0001 (7)	0.0039 (6)
C16	0.0513 (12)	0.0412 (10)	0.0350 (9)	0.0146 (9)	−0.0007 (8)	0.0062 (8)
C10	0.0436 (10)	0.0357 (9)	0.0308 (8)	0.0095 (8)	0.0011 (8)	0.0014 (7)
C12	0.0593 (13)	0.0541 (12)	0.0352 (10)	0.0124 (10)	0.0019 (9)	0.0112 (9)
C17	0.0581 (13)	0.0356 (9)	0.0366 (9)	0.0177 (9)	0.0012 (9)	0.0050 (7)
C9	0.0456 (11)	0.0371 (9)	0.0358 (9)	0.0106 (8)	0.0040 (8)	0.0054 (7)
C5	0.0453 (11)	0.0463 (11)	0.0409 (10)	0.0058 (9)	−0.0069 (9)	0.0043 (8)
C22	0.0624 (14)	0.0549 (12)	0.0409 (11)	0.0214 (11)	−0.0019 (10)	0.0099 (9)
C25	0.0541 (12)	0.0369 (9)	0.0306 (9)	0.0073 (9)	−0.0022 (8)	0.0062 (7)
C4	0.0460 (11)	0.0349 (9)	0.0349 (9)	0.0039 (8)	0.0063 (8)	0.0034 (7)
C15	0.0581 (13)	0.0408 (11)	0.0420 (11)	0.0018 (9)	0.0013 (10)	0.0070 (8)
C13	0.0632 (15)	0.0609 (14)	0.0367 (10)	0.0113 (12)	−0.0076 (10)	−0.0032 (10)
C11	0.0499 (12)	0.0375 (10)	0.0362 (9)	0.0035 (8)	0.0011 (8)	0.0049 (8)
C14	0.0661 (15)	0.0468 (12)	0.0502 (12)	−0.0016 (11)	−0.0082 (11)	−0.0059 (10)
C3	0.0578 (13)	0.0316 (9)	0.0534 (12)	0.0043 (9)	0.0090 (10)	−0.0021 (8)
C21	0.090 (2)	0.0736 (17)	0.0475 (13)	0.0376 (15)	0.0040 (13)	0.0240 (12)
C2	0.0689 (15)	0.0333 (10)	0.0554 (12)	0.0172 (10)	0.0163 (11)	0.0099 (9)
C18	0.0597 (14)	0.0463 (12)	0.0552 (13)	0.0176 (10)	0.0010 (11)	0.0091 (10)
C1	0.0510 (12)	0.0446 (11)	0.0412 (10)	0.0192 (9)	0.0118 (9)	0.0144 (8)
C19	0.0729 (17)	0.0464 (12)	0.0751 (17)	0.0147 (12)	0.0211 (14)	0.0150 (12)
C20	0.101 (2)	0.0574 (14)	0.0624 (15)	0.0338 (15)	0.0275 (15)	0.0264 (12)
C23	0.182 (5)	0.104 (3)	0.122 (3)	0.057 (3)	0.067 (3)	0.080 (3)
C24	0.283 (8)	0.101 (3)	0.174 (5)	0.071 (4)	0.070 (5)	0.086 (4)

### Geometric parameters (Å, º)

**Table:**

Br1—C1	1.886 (2)	C5—H5B	0.9700
O1—C4	1.365 (3)	C22—C21	1.382 (3)
O1—C5	1.418 (3)	C22—H22	0.9300
O2—C16	1.426 (2)	C4—C3	1.386 (3)
O2—N1	1.467 (2)	C15—C14	1.388 (3)
N1—C10	1.435 (2)	C15—H15	0.9300
N1—C7	1.485 (2)	C13—C14	1.365 (4)
C6—C25	1.469 (3)	C13—H13	0.9300
C6—C5	1.524 (3)	C11—H11	0.9300
C6—C7	1.536 (2)	C14—H14	0.9300
C6—C16	1.569 (3)	C3—C2	1.363 (3)
C8—C9	1.389 (3)	C3—H3	0.9300
C8—C4	1.393 (3)	C21—C20	1.376 (4)
C8—C7	1.505 (2)	C21—H21	0.9300
N2—C25	1.130 (3)	C2—C1	1.384 (3)
C7—H7	0.9800	C2—H2	0.9300
C16—C17	1.495 (3)	C18—C19	1.381 (3)
C16—H16	0.9800	C18—H18	0.9300
C10—C15	1.377 (3)	C19—C20	1.382 (4)
C10—C11	1.381 (3)	C19—H19	0.9300
C12—C13	1.375 (3)	C20—C23	1.516 (4)
C12—C11	1.376 (3)	C23—C24	1.371 (6)
C12—H12	0.9300	C23—H23A	0.9700
C17—C18	1.381 (3)	C23—H23B	0.9700
C17—C22	1.382 (3)	C24—H24A	0.9600
C9—C1	1.379 (3)	C24—H24B	0.9600
C9—H9	0.9300	C24—H24C	0.9600
C5—H5A	0.9700		
			
C4—O1—C5	114.15 (15)	O1—C4—C3	116.29 (18)
C16—O2—N1	102.54 (13)	O1—C4—C8	122.97 (17)
C10—N1—O2	107.73 (14)	C3—C4—C8	120.7 (2)
C10—N1—C7	115.95 (15)	C10—C15—C14	119.2 (2)
O2—N1—C7	99.71 (13)	C10—C15—H15	120.4
C25—C6—C5	109.23 (16)	C14—C15—H15	120.4
C25—C6—C7	111.41 (16)	C14—C13—C12	119.8 (2)
C5—C6—C7	108.42 (15)	C14—C13—H13	120.1
C25—C6—C16	114.63 (16)	C12—C13—H13	120.1
C5—C6—C16	110.13 (17)	C12—C11—C10	120.00 (19)
C7—C6—C16	102.74 (14)	C12—C11—H11	120.0
C9—C8—C4	118.41 (17)	C10—C11—H11	120.0
C9—C8—C7	120.38 (17)	C13—C14—C15	120.8 (2)
C4—C8—C7	120.82 (17)	C13—C14—H14	119.6
N1—C7—C8	115.44 (15)	C15—C14—H14	119.6
N1—C7—C6	98.84 (14)	C2—C3—C4	120.4 (2)
C8—C7—C6	112.34 (15)	C2—C3—H3	119.8
N1—C7—H7	109.9	C4—C3—H3	119.8
C8—C7—H7	109.9	C20—C21—C22	121.5 (2)
C6—C7—H7	109.9	C20—C21—H21	119.3
O2—C16—C17	109.47 (17)	C22—C21—H21	119.3
O2—C16—C6	104.02 (15)	C3—C2—C1	119.47 (19)
C17—C16—C6	116.39 (16)	C3—C2—H2	120.3
O2—C16—H16	108.9	C1—C2—H2	120.3
C17—C16—H16	108.9	C17—C18—C19	120.5 (2)
C6—C16—H16	108.9	C17—C18—H18	119.7
C15—C10—C11	120.04 (18)	C19—C18—H18	119.7
C15—C10—N1	123.18 (17)	C9—C1—C2	120.9 (2)
C11—C10—N1	116.75 (17)	C9—C1—Br1	119.56 (16)
C13—C12—C11	120.2 (2)	C2—C1—Br1	119.56 (15)
C13—C12—H12	119.9	C18—C19—C20	121.1 (3)
C11—C12—H12	119.9	C18—C19—H19	119.5
C18—C17—C22	118.8 (2)	C20—C19—H19	119.5
C18—C17—C16	121.91 (19)	C21—C20—C19	118.0 (2)
C22—C17—C16	119.3 (2)	C21—C20—C23	121.1 (3)
C1—C9—C8	120.11 (19)	C19—C20—C23	120.9 (3)
C1—C9—H9	119.9	C24—C23—C20	118.3 (4)
C8—C9—H9	119.9	C24—C23—H23A	107.7
O1—C5—C6	111.55 (17)	C20—C23—H23A	107.7
O1—C5—H5A	109.3	C24—C23—H23B	107.7
C6—C5—H5A	109.3	C20—C23—H23B	107.7
O1—C5—H5B	109.3	H23A—C23—H23B	107.1
C6—C5—H5B	109.3	C23—C24—H24A	109.5
H5A—C5—H5B	108.0	C23—C24—H24B	109.5
C17—C22—C21	120.2 (2)	H24A—C24—H24B	109.5
C17—C22—H22	119.9	C23—C24—H24C	109.5
C21—C22—H22	119.9	H24A—C24—H24C	109.5
N2—C25—C6	177.6 (2)	H24B—C24—H24C	109.5
			
C16—O2—N1—C10	−176.95 (16)	C7—C6—C5—O1	62.4 (2)
C16—O2—N1—C7	−55.56 (17)	C16—C6—C5—O1	174.11 (15)
C10—N1—C7—C8	−72.5 (2)	C18—C17—C22—C21	−0.7 (3)
O2—N1—C7—C8	172.22 (15)	C16—C17—C22—C21	177.9 (2)
C10—N1—C7—C6	167.50 (15)	C5—O1—C4—C3	−162.91 (18)
O2—N1—C7—C6	52.22 (15)	C5—O1—C4—C8	19.5 (3)
C9—C8—C7—N1	80.4 (2)	C9—C8—C4—O1	178.12 (18)
C4—C8—C7—N1	−106.9 (2)	C7—C8—C4—O1	5.2 (3)
C9—C8—C7—C6	−167.34 (17)	C9—C8—C4—C3	0.7 (3)
C4—C8—C7—C6	5.4 (3)	C7—C8—C4—C3	−172.24 (18)
C25—C6—C7—N1	−154.08 (15)	C11—C10—C15—C14	1.0 (3)
C5—C6—C7—N1	85.69 (17)	N1—C10—C15—C14	−177.1 (2)
C16—C6—C7—N1	−30.87 (17)	C11—C12—C13—C14	1.2 (4)
C25—C6—C7—C8	83.6 (2)	C13—C12—C11—C10	−1.8 (3)
C5—C6—C7—C8	−36.6 (2)	C15—C10—C11—C12	0.7 (3)
C16—C6—C7—C8	−153.14 (16)	N1—C10—C11—C12	178.96 (19)
N1—O2—C16—C17	158.99 (16)	C12—C13—C14—C15	0.6 (4)
N1—O2—C16—C6	33.95 (19)	C10—C15—C14—C13	−1.7 (4)
C25—C6—C16—O2	119.69 (18)	O1—C4—C3—C2	−179.65 (19)
C5—C6—C16—O2	−116.68 (17)	C8—C4—C3—C2	−2.0 (3)
C7—C6—C16—O2	−1.34 (19)	C17—C22—C21—C20	1.0 (4)
C25—C6—C16—C17	−0.8 (3)	C4—C3—C2—C1	0.9 (3)
C5—C6—C16—C17	122.83 (19)	C22—C17—C18—C19	0.3 (3)
C7—C6—C16—C17	−121.83 (18)	C16—C17—C18—C19	−178.3 (2)
O2—N1—C10—C15	20.2 (3)	C8—C9—C1—C2	−3.0 (3)
C7—N1—C10—C15	−90.5 (2)	C8—C9—C1—Br1	177.82 (14)
O2—N1—C10—C11	−158.00 (17)	C3—C2—C1—C9	1.6 (3)
C7—N1—C10—C11	91.4 (2)	C3—C2—C1—Br1	−179.19 (17)
O2—C16—C17—C18	−47.1 (3)	C17—C18—C19—C20	−0.1 (4)
C6—C16—C17—C18	70.4 (3)	C22—C21—C20—C19	−0.9 (4)
O2—C16—C17—C22	134.4 (2)	C22—C21—C20—C23	179.7 (3)
C6—C16—C17—C22	−108.1 (2)	C18—C19—C20—C21	0.4 (4)
C4—C8—C9—C1	1.8 (3)	C18—C19—C20—C23	179.8 (3)
C7—C8—C9—C1	174.74 (17)	C21—C20—C23—C24	−49.4 (7)
C4—O1—C5—C6	−54.0 (2)	C19—C20—C23—C24	131.2 (5)
C25—C6—C5—O1	−59.2 (2)		

### Hydrogen-bond geometry (Å, º)

*Cg*3, *Cg*4 and *Cg*5 are the centroids of the C1–C4/C8/C9, C10–C15 and C17–C22 rings, respectively.

**Table:**

*D*—H···*A*	*D*—H	H···*A*	*D*···*A*	*D*—H···*A*
C11—H11···O1^i^	0.93	2.57	3.475 (2)	165
C3—H3···*Cg*5^ii^	0.93	2.78	3.491 (3)	134
C5—H5*B*···*Cg*3^i^	0.97	2.86	3.730 (4)	149
C22—H22···*Cg*4^iii^	0.93	2.95	3.628 (3)	131

Symmetry codes: (i) −*x*+1, −*y*+1, −*z*+1; (ii) *x*, *y*+1, *z*; (iii) −*x*+1, −*y*, −*z*+1.
